# High-resolution imaging system for integration into intelligent noncontact total body scanner

**DOI:** 10.1117/1.JBO.30.9.096001

**Published:** 2025-09-08

**Authors:** Lennart Jütte, Sandra González-Villà, Josep Quintana, Rafael Garcia, Bernhard Roth

**Affiliations:** a Leibniz University Hannover, Hannover Centre for Optical Technologies, Hannover, Germany; b Coronis Computing S.L., Girona, Spain; c Universitat de Girona, Institute of Computer Vision and Robotics Research, Girona, Spain; d Leibniz University Hannover, Cluster of Excellence PhoenixD, Hannover, Germany

**Keywords:** melanoma diagnostics, noncontact dermoscopy, skin lesion imaging, total body dermoscopy, focus stacking, artificial intelligence

## Abstract

**Significance:**

Melanoma’s rising incidence demands automatable high-throughput approaches for early detection such as total body scanners, integrated with computer-aided diagnosis. High-quality input data is necessary to improve diagnostic accuracy and reliability.

**Aim:**

This work aims to develop a high-resolution optical skin imaging module and the software for acquiring and processing raw image data into high-resolution dermoscopic images using a focus stacking approach. The obtained hyperfocus images should significantly enhance the diagnostic performance of total body scanners in clinical settings.

**Approach:**

We employed focus stacking to merge multiple images, each with a limited depth of field, into a single hyperfocus image, ensuring every part of the skin is in clear focus. The method was implemented in the high-resolution imaging module using an electrically tunable liquid lens to quickly capture a series of differently focused images *in vivo*. Algorithms were developed for image alignment, focus measurement, and fusion, with the addition of deep learning–based super-resolution techniques to further enhance image quality. A classification model was trained to provide an artificial intelligence (AI)-based lesion classification.

**Results:**

The developed optical imaging system successfully produced noncontact dermoscopic images with complete focus across all skin topographies. The hyperfocus images obtained demonstrated high resolution of 28  μm and captured focus stacks at 50 frames per second, ensuring rapid acquisition and patient comfort, however, with some variance in resolution of individual lesions compared with contact-based dermoscopy standards.

**Conclusions:**

The focus stacking–based approach for noncontact dermoscopy improves the quality of diagnostic images by ensuring an all-in-focus view of differently shaped skin lesions, essential for early melanoma detection. Although the approach marks a substantial improvement in noninvasive skin imaging, the use of super-resolution techniques requires careful consideration to avoid compromising the authenticity of the raw data. This work enables the usage of advanced imaging and AI techniques in total body scanners for early melanoma detection in clinical practice.

## 1 Introduction

Melanoma represents a critical public health concern due to rising incidence rates globally,^1^ underscoring the urgency for effective early detection methods. Noncontact total body scanners delivering dermoscopic images of skin lesions, enhanced by computer-aided diagnosis systems, could offer high-throughput, automatic scanning capabilities to identify potential melanomas at their earliest stages.^2^ However, the quality of the dermoscopic images strongly affects the overall diagnostic performance of total body scanners^3^ and their acceptance in clinical practice. Unfocused images make the assessment by dermatologists more challenging and potentially less precise.^4^ The automatic skin cancer classification in noncontact mode is an active field of research.^5^ Regular total body skin screening procedures are the foundation for detecting melanomas in their early stages. After the surgical excision of an early-stage melanoma, the patient is considered healed. Currently, contact-mode dermoscopes are clinically established for the initial lesion analysis. Compared with the naked eye, a dermoscope can reveal information on subcutaneous structures with high resolution.^6^ Based on that, dermatologists decide upon excision of a suspicious lesion according to the ABCDE rule, the latter standing for asymmetry, border, color, diameter, and evolution of nevi.^7^ So far, the clinical gold standard for the diagnosis of melanoma is the histopathology, which involves the microscopic examination of excised skin tissue samples. The samples are manually stained and analyzed for characteristic signs of melanoma, such as irregular cell structures and abnormal melanin distribution.^8^

Pressing a contact-mode dermoscope against the skin, which is currently routine in skin examination, poses the risk of infections and distorts the skin geometry and color.^9^^,^^10^ Noncontact dermoscopy eliminates these problems and is thus advantageous compared with contact-based dermoscopy.^9^ With the help of a suitable optical imaging system, including liquid lenses with electrically tunable focus, the skin is in its natural state when imaged. Detailed visualization of skin lesions without physical contact represents a significant advancement in dermatology. One key innovation is the use of high-resolution cameras capable of capturing detailed images of the skin. Such high-resolution images provide enhanced visualization of skin structures, aiding in the accurate diagnosis of various skin conditions. In addition, the remote accessibility enables dermatologists to evaluate patients from a distance, reducing the risk of cross-infections, which is, for example, essential in managing contagious skin diseases and during pandemics.

A liquid lens is an optical component that manipulating the light field using different shapes of its surface. It is possible to rapidly modulate its focal length by electrical control.^11^ A downside to noncontact dermoscopy can be that for lesion topographies with a vertical extension larger than the depth of field (DOF) of the imaging system, the skin area under study is not always completely in focus.^12^^,^^13^ This can lead to a blur of the skin areas that are not within the DOF, complicating lesion assessment. In dermoscopy, a reliable diagnosis would be prevented by this limitation.

This work reports on the development of a high-resolution optical imaging unit and the implementation of software for the acquisition of the raw image data and its processing into a high-resolution dermoscopy image, denoted as a hyperfocus image. The main steps of the image processing pipeline are the acquisition of the raw data, the data denoising, the image alignment, the measurement of the image area, which is in focus, and, finally, the image stack fusion into the hyperfocus image. The resolution of the hyperfocus images can further be improved by the implementation of super-resolution algorithms based on deep learning.^14^ The developed system is capable of achieving a resolution of 28  μm, enabling detailed visualization of small skin features. The developed classification model shows a false-negative rate below 5%, which is considered a good result, indicating that the model performs adequately in minimizing missed melanoma diagnoses. Color calibration resulted in a reduction of ∼47% in the color distance between the raw data and the standardized color patches in the CIELab space. Finally, the HRIM captures a focus stack of a lesion at ∼50 frames per second (FPS), enabling rapid acquisition times below 5 s and ensuring maximum patient comfort.

## 2 Methods

### 2.1 High-Resolution Imaging Unit

**Fig. 1:**
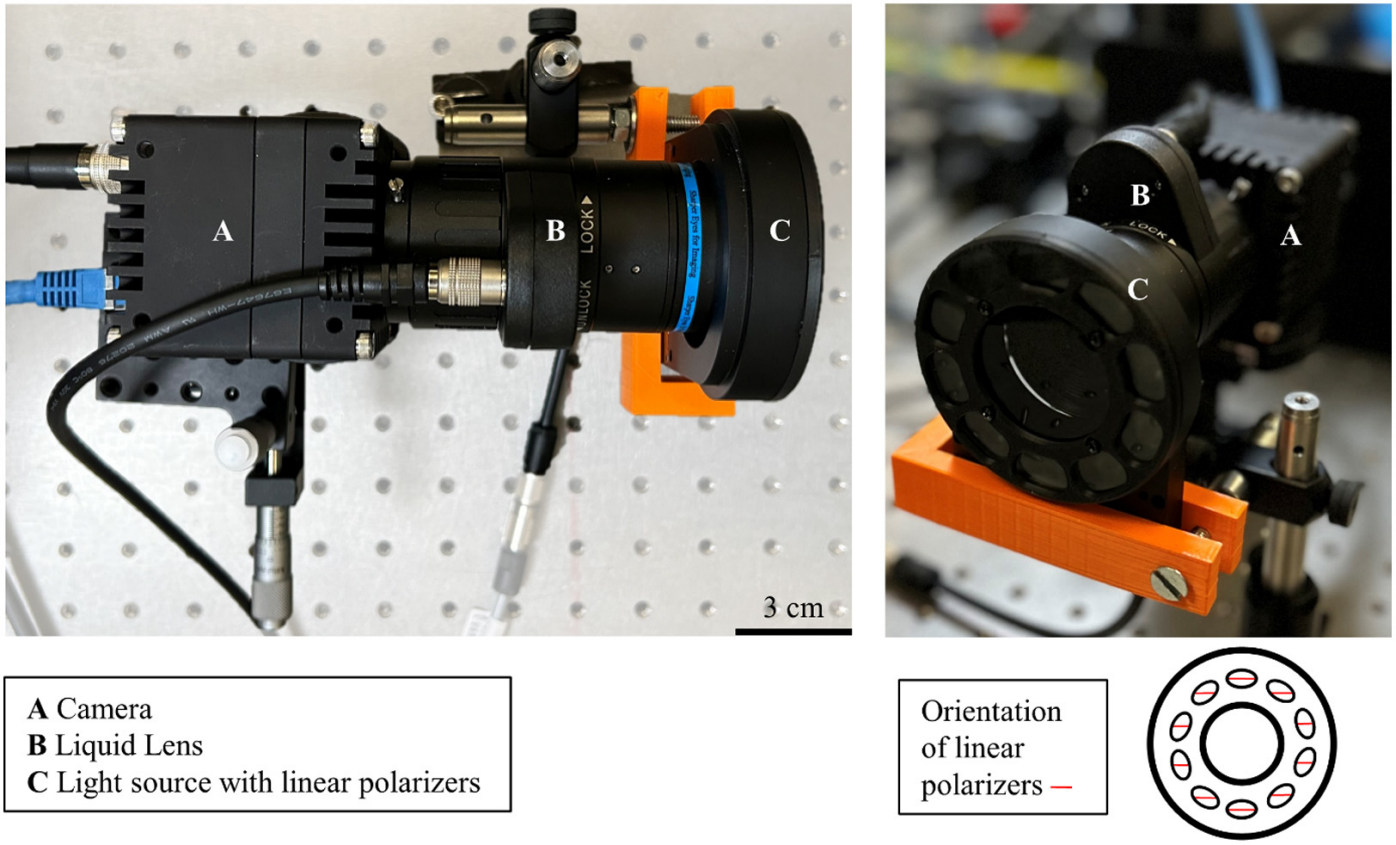
Photographs of the simple and robust noncontact dermoscope developed with (A) camera, (B) liquid lens, and (C) light source with 10 LEDs and polarizer elements, respectively, as main components. Left panel: top view. Right panel: front view. The orientation of the 10 linear polarizers is indicated in the bottom part. The light source contains an additional polarizer in cross-polarized configuration (with respect to the polarizers in front of the LEDs).

The setup of the noncontact dermoscope developed in this work is shown in Fig. 1. The system is very simple, robust, and easy to operate and consists of standard components, which are important for *in vivo* measurement and establishment of such systems in clinical environments in the future. Details on the optical system and its operation can be found in Refs. 9 and 13. Here, we briefly describe the main aspects for the sake of completeness.

For the illumination of the skin, a setup consisting of a custom-designed ring with 10 light-emitting diodes (LEDs) and 10 film-based linear polarization filters (ALB0810A and VPC antiglare filter, respectively, DCM SISTEMES, Valencia, Spain) (B) is used. The emitted light from the LEDs propagates through the polarizers and has a linear polarization afterward. The imaging part contains an additional polarizer in the imaging path, which is integrated in the light source, a liquid lens [EL-16-40-TC (5D), OPTOTUNE AG, Dietikon, Switzerland] (C), and a charge-coupled device camera (ORX-10GS-32S4C-C, FLIR Integrated Imaging Solutions Inc., Richmond, British Columbia, Canada) (A). The light that is scattered in deeper layers of the skin changes its polarization, whereas the part reflected at the surface maintains its polarization.^15^ Therefore, the polarizer filters the surface reflections in a cross-polarization configuration of the imaging polarizer and the light source polarizers.^15^ The EL-16-40-TC (5D) is an electrically tunable liquid lens designed for rapid and precise optical adjustments with a response time of 5  μs and a settling time of 25  μs.^16^ The operating principle of the liquid lens is based on electrostatic pressure. By applying an electric current to this shape-changing polymer lens, its refractive power can be controlled within milliseconds over a diopter range of −2  dpt to +3  dpt. This, in turn, corresponds to a certain focal plane accessible to the imaging system. The lens features a clear aperture of 16 mm and includes an integrated temperature sensor for *in situ* compensation of temperature effects, ensuring good focus stability and repeatability of about +/−0.05  dpt. This makes it ideal for applications requiring high imaging quality and a large beam diameter. In an initial version of the system, the tunability of the focal length and the precise focusing on the target skin area were verified by temporarily incorporating a precise infrared laser distance sensor (DT35-B15851, SICK AG, Waldkirch, Germany) into the setup. The adjustment of the focus with the liquid lens is based on the distance information provided by the distance sensor and a function derived from the calibration procedure. This procedure relies on the collection of value pairs of working distance and liquid lens current. During calibration, the liquid lens current was adjusted for different working distances until an imaged object was in focus. In general, the working distance is limited by the illumination intensity, the related signal-to-noise-ratio, and the desired image resolution (details on the liquid lens and the calibration procedure can be found in Ref. 13). With the automatic focus, it is possible to fully open the imaging aperture and accept a reduced DOF, because larger openings reduce the acquisition time due to shorter possible exposure time. The autofocus mechanism allows for a working distance of ∼45  cm with the ability to maintain focus at distances in a small range around the working distance (1 to 2 cm), confirming the reliable operation of the system. Finally, the image is captured by the FLIR Oryx 10GigE camera, model ORX-10GS-32S4C-C. This camera is equipped with a SONY IMX252 color sensor, offering a resolution of 3.2 MP and a frame rate of 216 FPS. The captured images are transferred via a high-speed 10GBASE-T interface, supporting transfer speeds of up to 10  Gbit/s. This allows for the capture of 12-bit images at 4K resolution and more than 60 FPS. The computer controls the camera and the liquid lens, by adjusting both the camera’s shutter and the lens’s diopter. The incoming frames are processed via a network card (MYRICOM, Inc., Arcadia, California, United States). The imaging units are designed to be used in a total body scanner currently in development.

**Fig. 2:**
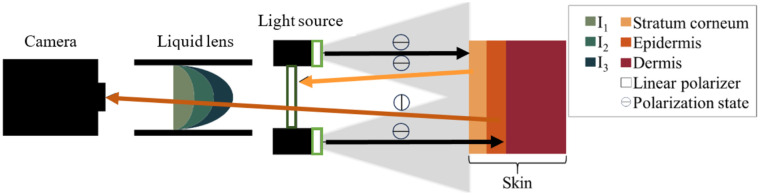
Sketch of noncontact dermoscope with the principle of cross-polarized skin imaging with a liquid lens. The arrows mark example trajectories of the light emitted by the LED light source propagating to the skin (black) and reflected from different layers of the skin (orange: from stratum corneum, dark orange: from epidermis). Gray cones indicate areas illuminated by individual LEDs. Open rectangles stand for the linear polarizers in front of the individual LEDs of the light source (light green) and in the imaging path (dark green), respectively. Lines in the open circles indicate the polarization state. Although light reflected from the epidermis is filtered by the linear polarizer in the imaging path, the light reflected from the epidermis is transmitted. $I_{1}$, $I_{2}$, and $I_{3}$ denote different current values applied to the liquid lens to tune the focal length.

Figure 2 illustrates the underlying principle of cross-polarized skin imaging. The linearly polarized light from the light source [i.e., 10 LEDs with polarizers] penetrates into deeper layers of the skin where its polarization is changed. Light that is directly reflected from the skin’s surface does not change its polarization and will not pass through the imaging polarizer in front of the liquid lens, as the latter is rotated by 90 deg relative to the polarization of the illumination. The cross-polarization configuration is well established in dermatology as it reduces the amount of surface glare and improves the visibility of miniscule lesion patterns relevant for lesion classification.^15^

The estimated cost of the hardware components for the high-resolution imaging module (HRIM) totals ∼6000  €. The camera module is priced at 2000 €. The light source, which provides appropriate polarized LED illumination, costs ∼500  €. A liquid lens, essential for adaptive focus stacking and precise imaging, is priced at 1500 €. The high-speed network card, required for real-time data transfer and processing, costs ∼1500  €, whereas custom mounts, enclosures, and optical housings for the secure integration of all components are priced at 500 €. Economically, the cost-effectiveness of the prototype is an important consideration.

### 2.2 Data Acquisition

**Fig. 3:**
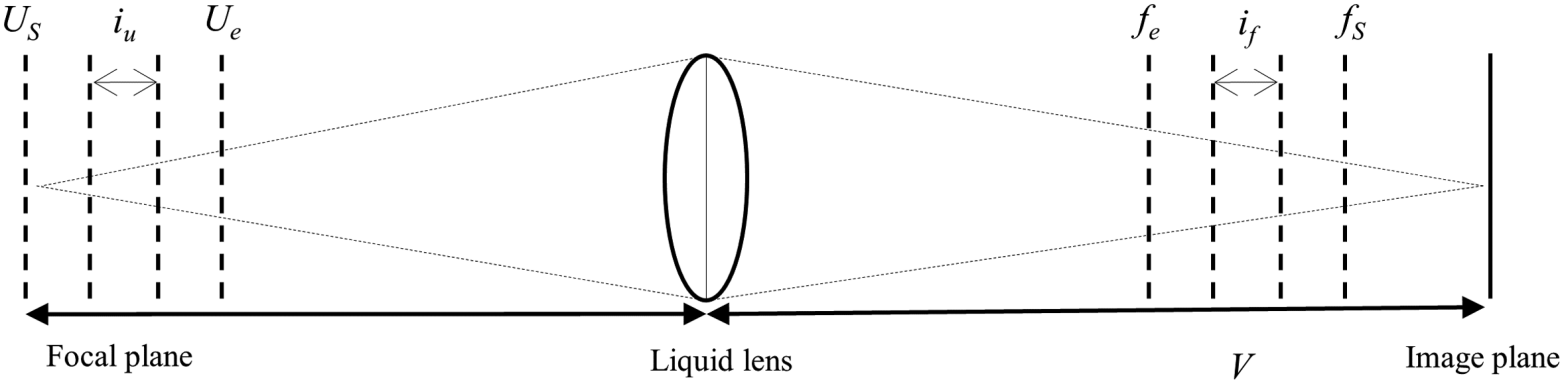
Sketch of the thin lens model with a liquid lens. The focal length of the liquid lens changes from $f_{s}$ to $f_{e}$ with an interval $i_{f}$. Correspondingly, the focal plane moves from $U_{s}$ to $U_{e}$.

In the following, the basics for the image acquisition are described. Figure 3 sketches the optics for collecting a stack of images from different focal planes using a thin lens model.

**Fig. 4:**
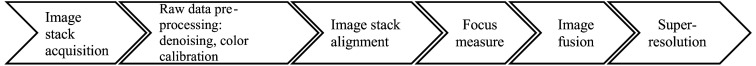
Sketch of the main steps of the image processing pipeline for our imaging system and the future total body scanner consisting of several such imaging systems. The raw data preprocessing step includes denoising and color calibration. The image stack alignment, the focus measure, and the image fusion are part of the focus stacking step. The generation of the super-resolution image constitutes the last step and creates the images to be used for skin lesion classification.

The focal length f can be calculated by the thin lens equation

$$\frac{1}{f}=\frac{1}{U}+\frac{1}{V}.$$ (1)

where U and V stand for the object distance and the image distance, respectively. The DOF of an image is limited, which means that for some skin topographies only parts of the lesion are in focus.^13^ To obtain an all-in-focus image of the lesion, the focused regions from every captured image are extracted and stacked together. This method is called focus stacking.^17^ Figure 4 shows the workflow for focus stacking as implemented in this work.

Through stepwise changing of the current of the electromagnet in the liquid lens, differently focused images are captured in one sequence within less than 5 s. The correction of the colors constitutes the main aspect of raw data preprocessing, which is performed after denoising of the raw data. Because of the misalignment between the images of the stack, the images need to be aligned with respect to each other, ensuring that they are overlaid pixel by pixel. Extracting the in-focus areas from each image is called focus measure.^18^ The focus measure is calculated for every pixel in the stack to evaluate its focus. Based on the weights derived from the focus measure, the images are fused to create an all-in-focus image. The image stack acquisition was implemented in *Python* (version: 3.6.4) with the libraries *PyCapture2* for the camera and *Opto* for the liquid lens. For rapid and convenient capture, a graphical user interface was designed based on Qt (version: 5.15.4).

**Fig. 5:**
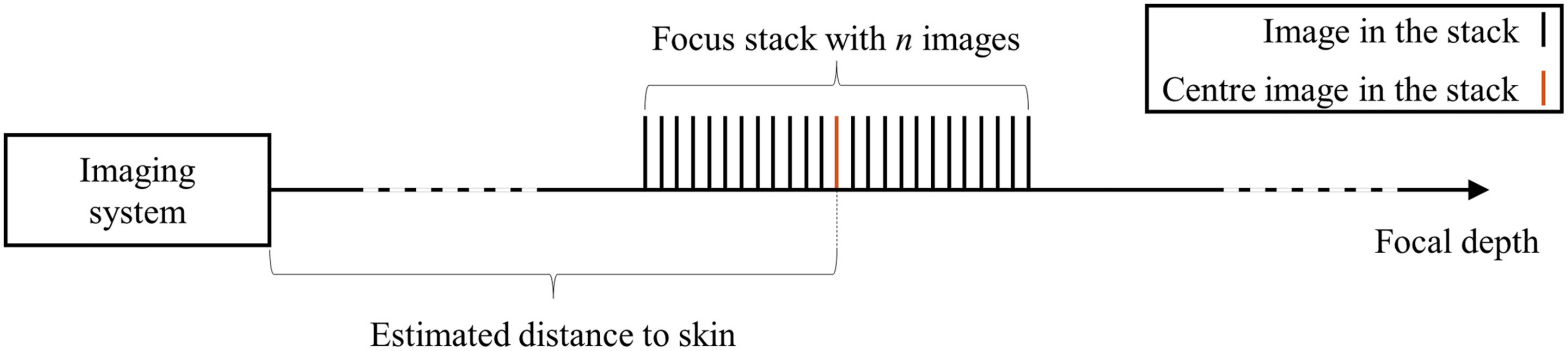
Schematic of the focus stacking process. A focus stack of n images is created around the estimated distance to the skin, accounting for sensor inaccuracies, skin topography, and patient movement.

To achieve focus stacking, in the current approach, the distance to the patient is initially measured using a distance sensor (LiDAR camera L515, Intel RealSense, Intel Corporation, Santa Clara, California, United States). Due to potential inaccuracies in the sensor and variations in skin topography, as well as patient movement during image acquisition, the focus stack includes images captured at the estimated distance and at distances both slightly before and after this estimated position, as displayed in Fig. 5. This approach ensures that the focal depth adequately covers the target area. The stack consists of n images (typically between 10 and 20), providing a comprehensive set of focused images across the estimated range determined by the size and shape of the image’s skin area. This is to ensure that all parts of a particular skin area are in focus in at least one of the images.

### 2.3 Raw Data Denoising

Noise from the CMOS sensor chip impacts imaging quality and system functionality. The typical types of noise encountered in image acquisition are photon noise, thermal noise, readout noise, fixed pattern noise, and gain noise. Photon noise arises from the uncertainty in the incident photon flux. The average number of collected photons varies, causing inherent noise equal to the square root of the signal.^19^ Photon noise is especially problematic in low-light imaging conditions or when capturing fine skin structures. If left uncorrected, it can reduce the image contrast, complicating the differentiation between healthy and malignant regions of skin lesions and affecting the accuracy of both artificial intelligence (AI) models and dermatological assessments. Thermal noise is light-independent. This noise is due to thermal energy affecting the sensor’s charge build-up. It becomes more significant during long imaging sessions or in higher-temperature environments, where heat can distort critical image details.^20^ In dermoscopic imaging, thermal noise can blur small, subtle features, making it harder to identify fine lesion borders. It is temperature-dependent and mitigated using Peltier cooling. Readout noise occurs during the conversion of electrons to a voltage signal. It is following a Gaussian distribution with similar fluctuations across all pixels.^21^ This type of noise is particularly relevant when trying to capture high-resolution images as it affects image sharpness, in particular, in low-light regions or areas of fine detail. If a camera images a uniformly illuminated featureless target, any deviations are due to fixed pattern noise, appearing as bright and dark pixel patterns.^22^ It is measured as the difference between maximum and minimum pixel values. In dermoscopic imaging, fixed pattern noise can distort the uniform appearance of the skin surface, leading to artifacts that might be mistaken for true skin features. Reducing this noise is vital for achieving consistent image quality, especially in automated analysis.

The image noise must be removed (or reduced) to improve the image quality and avoid the propagation of artifacts that could be enhanced along the image processing pipeline. Therefore, denoising should be implemented at an early stage in each image processing pipeline. In this work, denoising is performed as the first pre-processing step on the raw data.

Nonlocal means (NLM) filters work by averaging the values of pixels in an image based on the similarity of their surrounding neighborhoods, rather than just their spatial proximity. This approach helps preserve important image details, such as edges while effectively reducing noise.^23^ In this work, the implemented denoising NLM filter is a build-in function of MATLAB (MathWorks, Inc., Natick, Massachusetts, United States). For each pixel in the image, the filter compares a patch centered on that pixel with patches centered on other pixels within a search window. The similarity between two patches is calculated based on a weighted Euclidean distance. Patches that are more similar (i.e., their pixel values are closer to each other) are given higher weights. Each pixel in the output image is then replaced by a weighted average of the pixels in its search window. The weights are determined by the similarity measure calculated in the previous step. The MATLAB function *imnlmfilt* implements this algorithm, whereas the *DegreeOfSmoothing* controls the amount of smoothing, where higher values result in more smoothing. The *SearchWindowSize* is the size of the window used to search for similar patches, and the *ComparisonWindowSize* is the size of the patches used for comparison. By adjusting these parameters, the denoising process can be fine-tuned to achieve the desired balance between noise reduction and preservation of image details.

### 2.4 Color Calibration

The goal of color calibration is to ensure that image colors closely match the natural colors.^12^ This process uses a standard reference target (Colorchecker classic, X-RITE, Grand Rapids, Michigan, United States), which provides calibrated color values. A raw image is captured for each color tile on the target. The color difference between these raw images and the reference values is minimized during calibration. The color difference can be objectively analyzed by the color distance, which is calculated as the Euclidean distance between two colors in the respective color space. The color distance between colors in the red green blue (RGB) color space can be calculated according to Eq. (2)

$$\text{Color distance}\;(R,G,B)=\sqrt[{2}]{(R_{1}-R_{2}{)}^{2}+(G_{1}-G_{2}{)}^{2}+(B_{1}-B_{2}{)}^{2}}.$$ (2)

In addition, the distance in the CIELab (CIE: International Commission on Illumination, L: perceptual lightness, and a and b: factors that describe the share of red, green, blue, and yellow) color space can be analyzed, which is designed to be as close as possible to the color difference perceived by a human.^24^^,^^25^ The color distance between two colors in the CIELab space can be calculated according to Eq. (3)

$$\text{Color distance}(L,a,b)=\sqrt[{2}]{(L_{1}-L_{2}{)}^{2}+(a_{1}-a_{2}{)}^{2}+(b_{1}-b_{2}{)}^{2}}.$$ (3)

In the following, the methods for color difference minimization used in this work—linear regression, nonlinear regression, and k-means clustering—are introduced. These methods were applied to improve the accuracy of color calibration by minimizing the difference between the observed experimental color values and the reference target values.

Linear regression is a statistical method that models the relationship between a dependent variable and one or more independent variables by fitting a linear equation to the observed data.^26^ In the context of color calibration, linear regression was used to map the experimental color values to the reference values provided by the reference target manufacturer. The goal is to find the best-fitting linear relationship that minimizes the difference between the predicted and actual reference values. Nonlinear regression extends linear regression by fitting a polynomial equation to the data.^27^ The quadratic regression method allows for more flexibility than linear regression by fitting curves to the data. A polynomial equation, including cubic terms, was used here to further capture complex nonlinear relationships. Finally, k-means clustering is an algorithm used to partition a dataset into a specified number of clusters (k).^28^ Each data point is assigned to the cluster with the nearest mean. The experimental color values were grouped into 2, 3, and 4 clusters. The centroid of each cluster represents the average color value, and these centroids were used to adjust the calibration function.

By comparing the performance of these different methods, i.e., by evaluating the resulting color distances in the RGB and CIELab color spaces, we identified the most accurate approach (resulting in the smallest color distance) for calibrating the colors for our system. These results, discussed further in Sec. 3.3, demonstrate how each method performed, ensuring that the experimental values closely match the reference values from the target.

### 2.5 Focus Stacking

After the pre-processing of the raw data, the image stack needs to be aligned. An unavoidable cause for misalignment between the frames is that objects at different distances are magnified differently. This is because of the change in the focal length between frames. Other reasons for misalignment between frames are potential movements of the patient or the system. An enhanced correlation coefficient (ECC)-^29^ based method was employed on the captured images to eliminate the misalignment from these two sources. Equation (4) uses the Euclidean norm to quantify the error between the reference image $I_{r}$ and the warped input image $I_{w}(p)$. The unknown parameters are denoted as p. The alignment problem is to estimate the parameter p. The most magnified image in the stack is used as the reference image. The other images of the stack are being correlated to the reference image sequentially. The ECC method aims to minimize $E_{\mathrm{ECC}}(p)$. An estimated affine transformation matrix is calculated by minimizing the difference between the reference image and the warped input image

$$E_{\mathrm{ECC}}(p)||\frac{I_{r}}{\left|I_{r}\right|}-\frac{I_{w}(p)}{\left||I_{w}(p)|\right|}|{|}^{2}.$$ (4)

After the image stack alignment, the focused areas of each image are calculated. The focus measure is subsequently used as a weight for the image fusion step. The focus measure is calculated using the Laplacian variance method.^30^ This method involves applying a Laplacian filter to each image to highlight regions of rapid intensity change (edges and fine details). The variance of the filtered image is then computed within a local window, providing a quantitative measure of focus. Higher variance indicates sharper, more in-focus regions.

The focus value $f_{m,i}$ of a pixel in the input image i can be obtained by applying a focus measure to each input image.^31^ As described in Eq. (5), for each pixel (u,v), the value of the fused image $I_{\text{fused}}$ is the weighted sum of input images $I_{i}$. Here, the number of images in the stack is k

$$I_{\text{fused}}(u,v)=\overset{k}{\underset{i=1}{\sum }}\frac{f_{mi}(u,v)}{\sum_{i=1}^{k}f_{mi}(u,v)}I_{i}(u,v).$$ (5)

**Fig. 6:**
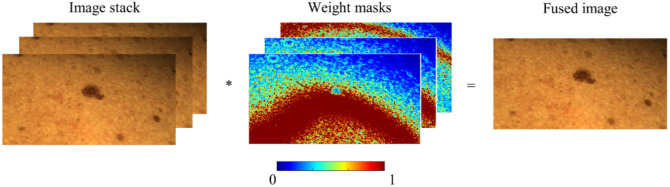
Visualization of the image fusion process. Fusion is achieved by assigning a weight to each pixel based on its focus value, with the fused image representing the weighted sum of all input images. The displayed weight masks were calculated using the Laplacian variance method, where red areas indicate regions of high focus.

Figure 6 visualizes the fusion process. The pixel on the fused image is a weighted sum of the pixels in the input image stack. The images used here and throughout this work are typical examples obtained from benign lesions of volunteers. They serve to demonstrate the capabilities of the system and the imaging pipeline. Overall, several 10 such image stacks from different lesions have been taken so far. Clinical measurements with the fully assembled total body scanner on benign and malignant lesions, including melanoma, are part of the next phase and are not the focus of this work.

To evaluate multifocus image fusion objectively, different metrics from four categories can be used.^32^ The metrics chosen in this work to validate the results from Sec. 3.4 are shown in Table 1; the procedure is described in detail in Ref. 13.

**Table 1:** Image fusion assessment metrics used in this work.

Metric	Category	Description
Normalized mutual information^33^	Information theory	Measures the distance between the fused image and the input image.
Spatial frequency^34^	Image feature	Assesses the first-order gradient error between the fused image and the input image in four directions.
Yang’s structural similarity index (SSIM)^35^	Image structural similarity	Evaluates the SSIM between the fused image and the input image.
Chen-Varshney index^36^	Human perception	Analyzes edge information, local region saliency, and similarity to gauge human perceptual quality.

### 2.6 Super-Resolution

Total body photography setups usually do not achieve the same resolution as digital dermoscopes.^37^ To mitigate the slight difference in resolution between the hyperfocus images obtained through focus stacking and the standard of contact dermoscopy, a super-resolution approach is implemented in this work. Super-resolution is a technique used to enhance the resolution of images, allowing for the generation of high-resolution images from low-resolution inputs. This deep learning approach is based on a generative adversarial method (GAN). For example, the generative adversarial network prior embedded network (GPEN) has been used for the restoration of blurry faces from degraded face images.^38^ In this study, a pre-trained super-resolution model (Deep AI Inc., San Francisco, California, United States), based on the super-resolution generative adversarial network (SRGAN) framework, is utilized. The SRGAN is a deep learning model designed to produce realistic high-resolution images using a combination of a generator and a discriminator network. To accomplish this, it employs a perceptual loss function composed of both an adversarial loss and a content loss.^39^

### 2.7 Objective Resolution Analysis with USAF target

The traditional method for assessing the resolution of noncontact dermoscopy systems involves the use of a USAF 1951 resolution target.^10^^,^^12^ This target comprises groups of line pairs with varying spacing, and the resolution is determined by visually inspecting which lines can be clearly distinguished. However, this method is inherently subjective, relying heavily on the observer’s judgment and experience, which can lead to inconsistent results and uncertainty.^40^

**Fig. 7:**
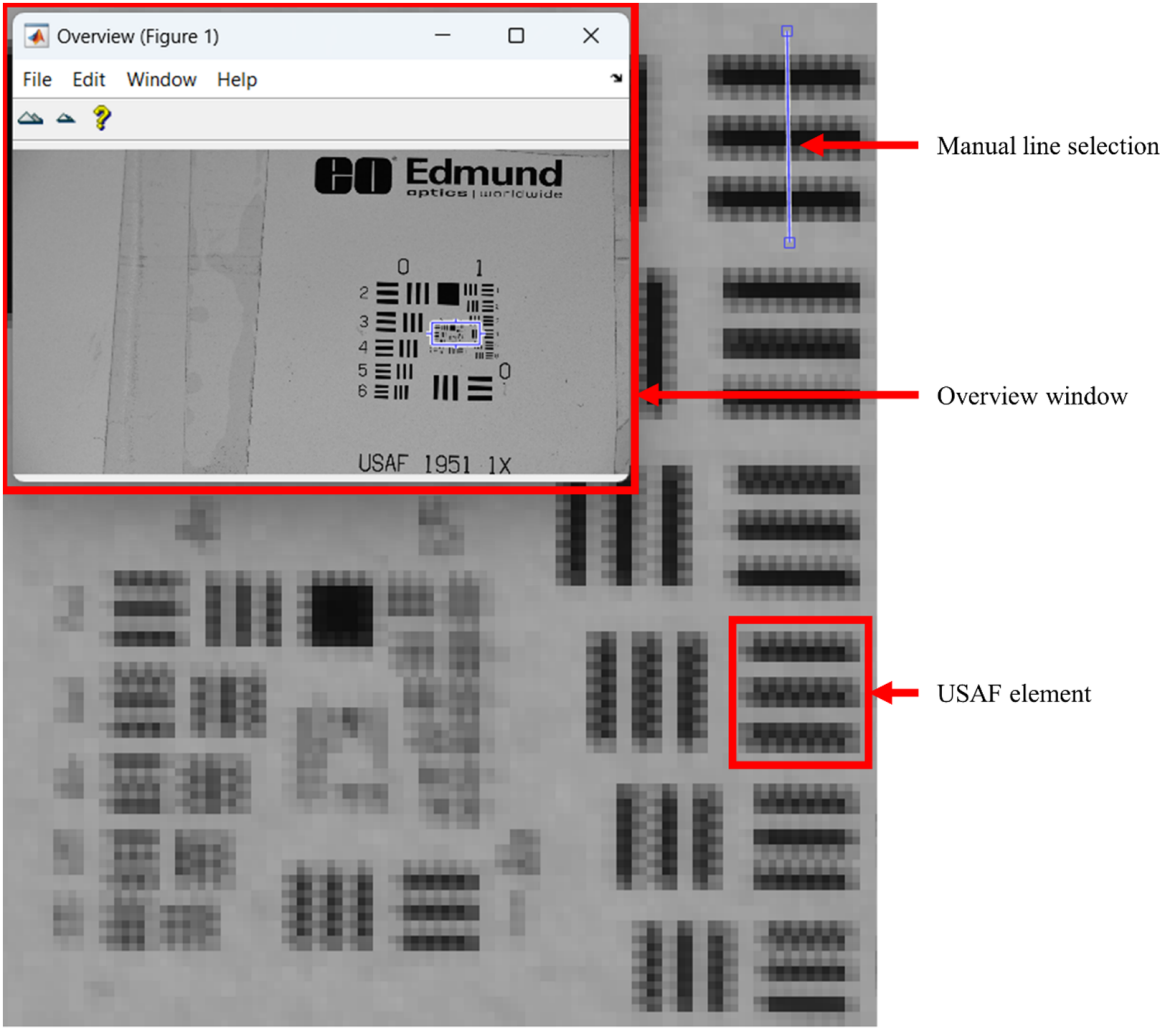
Semi-automated analysis of the USAF 1951 resolution target in MATLAB for comparing the resolution of skin imaging devices. Users manually select lines across the target elements for evaluation.

To address the subjectivity in the traditional subjective analysis of USAF 1951 targets, we developed an objective evaluation method using MATLAB. Our approach involves analyzing the intensity profiles of the USAF target elements and automating the detection of resolved elements. The algorithm is based on six main steps: image acquisition, line drawing, profile smoothing, peak detection, resolution criteria, and visualization. The USAF 1951 target image is captured using our noncontact dermoscopy system and converted to grayscale for analysis. The user is prompted to draw multiple lines across the elements of interest in the USAF 1951 target. For each line, the user confirms the position, and the intensity profile along the line is extracted. The intensity profile for each line is smoothed using the Savitzky–Golay filter to reduce noise and minor fluctuations.^41^ Significant maxima and minima in the smoothed profile are detected using the *findpeaks* function in MATLAB. The prominence of peaks is adjusted to filter out insignificant peaks. An element is considered resolved if there are three clear minima and two maxima between them, representing three black lines on a white background. In addition, the minima must fall below a threshold defined as 80% of the maximum intensity in the profile. The intensity profiles are plotted in subplots, with maxima and minima marked, and the resolution status of each element is displayed in the respective subplot. Figure 7 shows semi-automated resolution analysis realized using MATLAB.

The overview window in the top left is used to navigate the target. The user is prompted to select the endpoints of the blue line, which is then analyzed. The user selects a line that crosses the target element, where the element is still resolved. Subsequently, the user progresses through each element until the first element that is no longer resolved is identified.

### 2.8 Lesion Classification

AutoKeras is an open-source library designed to automate the process of developing and tuning deep learning models.^42^ It is built on top of Keras, which is a popular deep learning application programming interface (API) written in Python and capable of running on top of TensorFlow. AutoKeras aims to make deep learning more accessible to a broader user community by providing an easy-to-use interface for tasks such as image classification, text classification, and regression. It automates the process of finding the best model architecture and hyperparameters for a given task. This is achieved through a process called neural architecture search. It provides a high-level API that allows users to specify their machine learning task with minimal code. Users can quickly prototype models without deep knowledge of machine learning or neural networks. It supports a range of data types, including images, text, and structured data, making it versatile for different kinds of machine learning problems.

The *ImageClassifier* class in AutoKeras is designed specifically for image classification tasks. It automates the process of developing a deep learning model to classify images into predefined categories. With *ImageClassifier*, users can build image classification models with just a few lines of code. This reduces the complexity and time required to develop such models from scratch. The *ImageClassifier* handles common preprocessing steps such as resizing images, normalizing pixel values, and data augmentation, which are crucial for training robust image classification models. It automatically searches for the best neural network architecture and optimizes the hyperparameters to achieve the best performance on the given dataset. Users can easily train and evaluate the model using simple method calls. AutoKeras takes care of the underlying training loop, including data loading, batching, and model evaluation.

The AutoKeras model used in this work for classifying dermoscopic images of nevi and melanoma consists of several layers and transformations applied sequentially to the input data. The model begins with an input layer designed to accept images of 224 by 224 pixels in size with three color channels (RGB). The images are then cast to float32 format, followed by normalization to ensure the data is on a comparable scale. The model incorporates several data augmentation techniques, including random translation, random flipping, and random rotation, to enhance the robustness of the model by simulating variations in the training data. The core of the model is built around a pre-trained ResNet50 architecture, which is a well-established deep learning model known for its performance in image classification tasks.^43^ The ResNet50 layer processes the input data through its numerous convolutional and residual layers, resulting in a feature map of size 7 by 7 with 2048 channels. To reduce the dimensionality of these feature maps, a global average pooling layer is applied, producing a single vector of 2048 features. This condensed feature vector is then passed through a dense (fully connected) layer with a single unit, corresponding to the binary classification task of distinguishing between nevi and melanoma. The final output of the model is obtained through a classification head that applies an activation function, resulting in a single output value. This value indicates the predicted class of the input image.

The model was saved and compiled with the *Adam* optimizer and the *BinaryCrossentropy* loss function. It was trained with a batch size of 10, utilizing transfer learning from different models. A callback function was used during training to monitor the validation loss, ensuring that the model with the minimum validation loss was saved. This saved model was then tested on the testing data to evaluate its performance.

In this work, various skin disease datasets were analyzed. A custom dermoscopic dataset was created by aggregating images from multiple sources (PH2,^44^ ISIC,^45^ and Derm7pt^46^). After filtering, the dataset was cleaned to improve quality, which is crucial for model performance. The initial custom dataset included images from different sources. Melanomas and nevi were chosen to develop a dermoscopic skin disease classifier. Images of poor quality were manually filtered out, and the dataset was balanced to ensure an even representation of both types. The dataset was balanced by randomly selecting 2813 images for each class. To prevent bias, the balanced dataset was divided into training, validation, and testing sets, with 70% for training and 30% for testing. The final dataset consisted of 1952 images per class for training and 861 per class for testing. This dataset, though limited, has improved quality through rigorous cleaning, making it suitable for training a robust dermoscopic skin disease classifier.

## 3 Results

### 3.1 Imaging System Evaluation

**Fig. 8:**
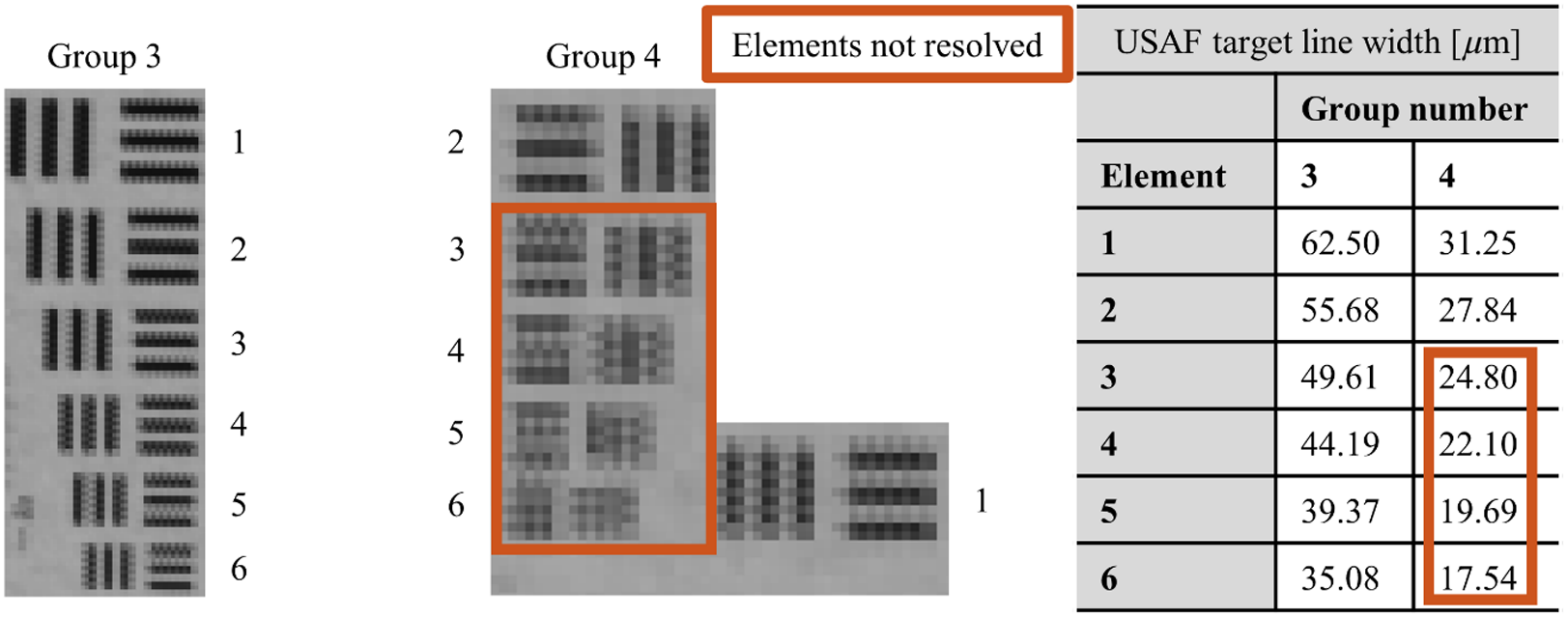
USAF 1951 target groups 3 (left) and 4 (center) captured at a working distance of 30 cm. All elements of group 3 and the first two elements of group 4 are resolved. The line width specifications for each group and element are shown on the right.

Figure 8 displays the crops of the USAF 1951 target for groups 3 and 4 acquired with the noncontact dermoscope under background illumination of an LED panel.

**Fig. 9:**
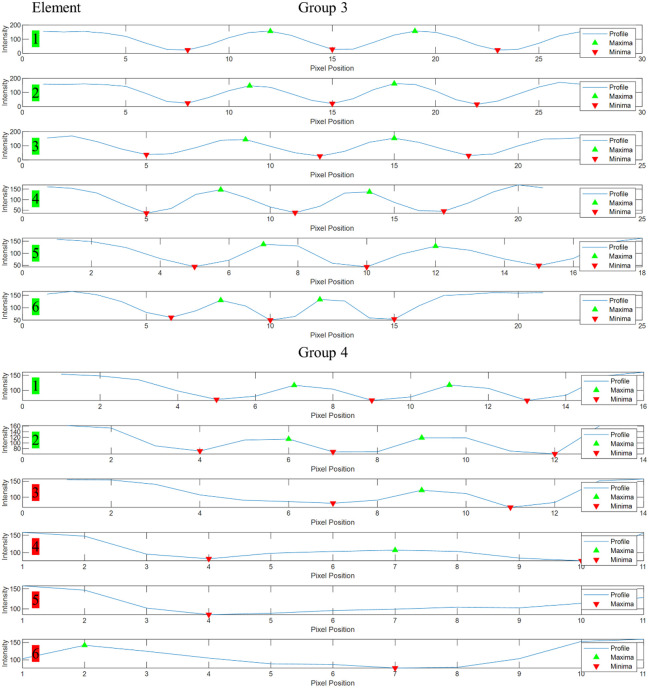
Intensity profiles for elements in groups 3 and 4 of the USAF 1951 target. Green element numbers indicate resolved elements, whereas red element numbers denote unresolved ones. Green triangles mark maxima, and red triangles indicate minima.

The developed algorithm was applied to objectively analyze the resolution of elements in groups 3 and 4 of the USAF 1951 target. In Fig. 9, the intensity profiles of each element are given, with maxima (green triangle) and minima (red triangle) indicated. The color of the element number displays whether the element is resolved according to the objective criteria (green: resolved; red: not resolved).

In Fig. 9, the green triangles represent the detected maxima, and the red inverted triangles represent the detected minima. The results indicate that the algorithm can objectively determine the resolution status of each element, providing a consistent and reproducible assessment. All elements in group 3 and the first two elements of group 4 were found to be resolved, demonstrating the effectiveness of the objective evaluation method. The analysis of the USAF 1951 target reveals that the developed HRIM can resolve features as small as 28  μm, representing a 10.9% improvement in detail resolution compared with an objective assessment conducted with the prototype by Fricke et al.^12^ The horizontal and vertical field of view (FOV) is 8.6 and 4.5 cm, respectively, representing a 17.5-fold increase compared with the prototype described in Ref. 12.

### 3.2 Raw Data Denoising

**Fig. 10:**
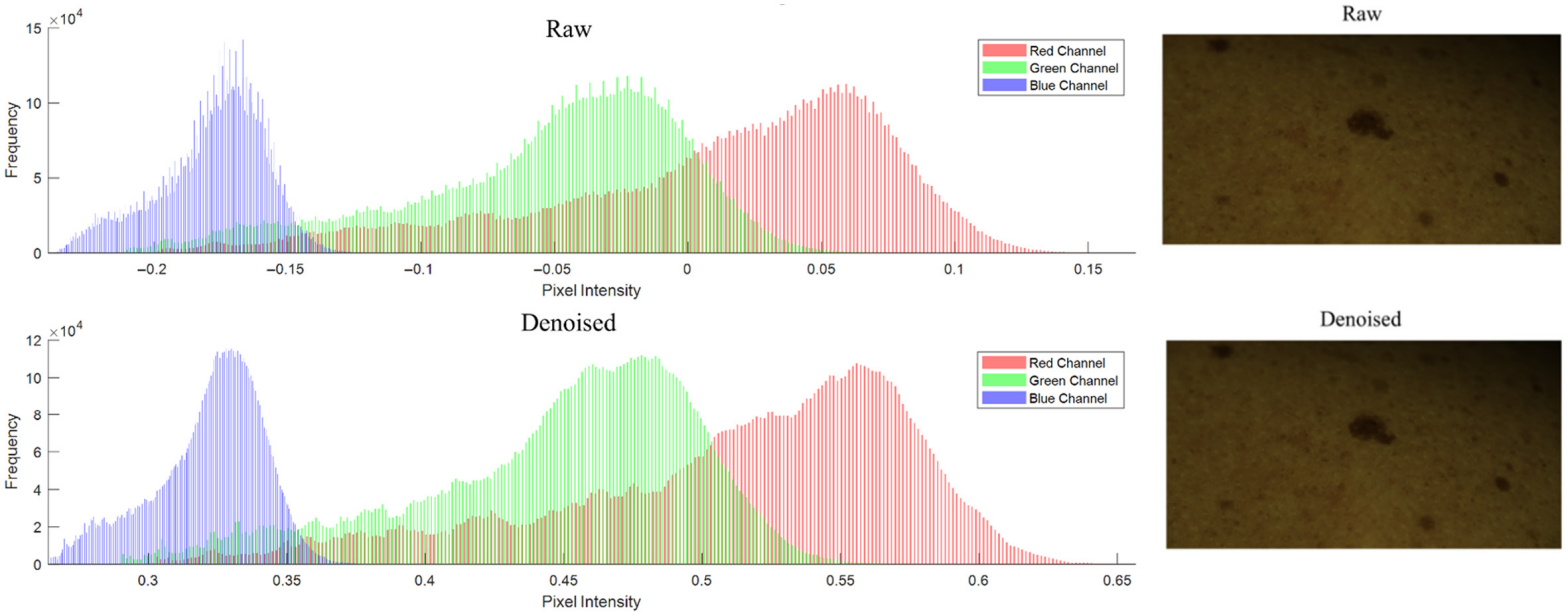
Left (top and bottom) panels: Comparison of raw data histograms for color channels before (raw) and after denoising. Each of the displayed histograms is cropped from the original size of the histograms. Right (top and bottom panels): Raw input image crop before and after denoising. The images appear dark due to the nature of the raw data, where the acquisition time is low and potential over-exposure is being avoided.

The implementation of the raw data denoising described in Sec. 2.3 on the raw image stack data is illustrated in Fig. 10. Due to the nature of the raw image data, the effect of the denoising is not obvious by visual inspection, see images in right (top and bottom) panels in Fig. 10. Therefore, we compare the histograms of the original image and the denoised image for each color channel, left (top and bottom) panels in Fig. 10. From there, it is obvious that the denoising results in a smoothing of the histograms for each color channel. To quantitatively assess the effectiveness of the denoising process, we performed a line profile analysis on a segment of the image.

**Fig. 11:**
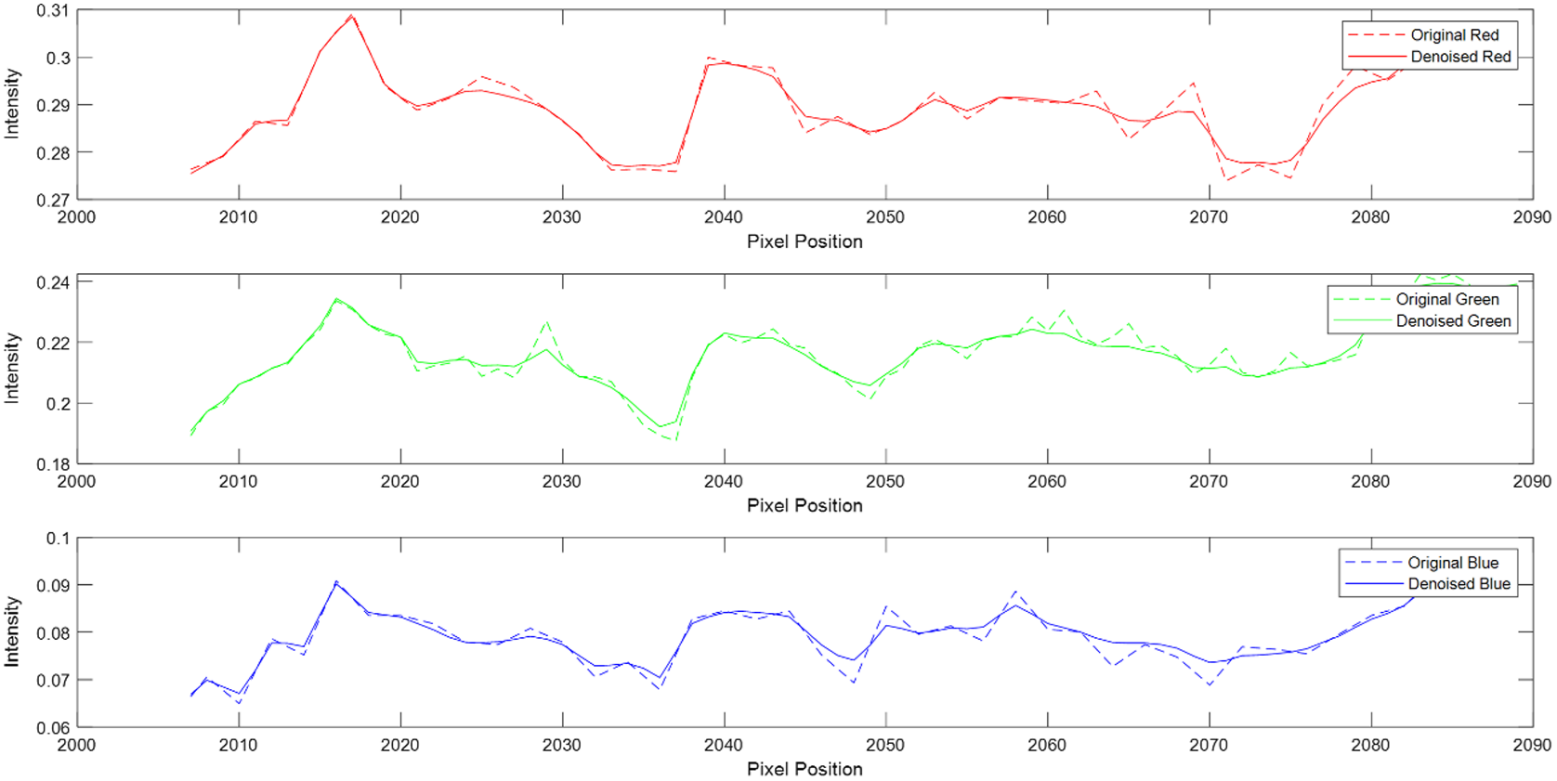
Line profile comparison. The dashed lines show the line profiles of the original noisy image, and the solid lines represent the line profiles of the denoised image.

We extracted line profiles from the central horizontal line of the image for each color channel (red, green, and blue), focusing on a segment that is 2% of the image width, centered around the middle of the image. The profiles for the original noisy image (dashed lines) and the denoised image (solid lines) are plotted for each color channel in Fig. 11. The line profile analysis demonstrates that the nonlocal means filter significantly reduces noise across all color channels, resulting in smoother intensity profiles. This analysis confirms that the denoising process preserves important image details while effectively removing noise, thus enhancing the overall image quality. This suggests that the denoising approach is adequate for improving overall image quality without compromising the accuracy of subsequent diagnostic analysis.

### 3.3 Color Calibration

**Fig. 12:**
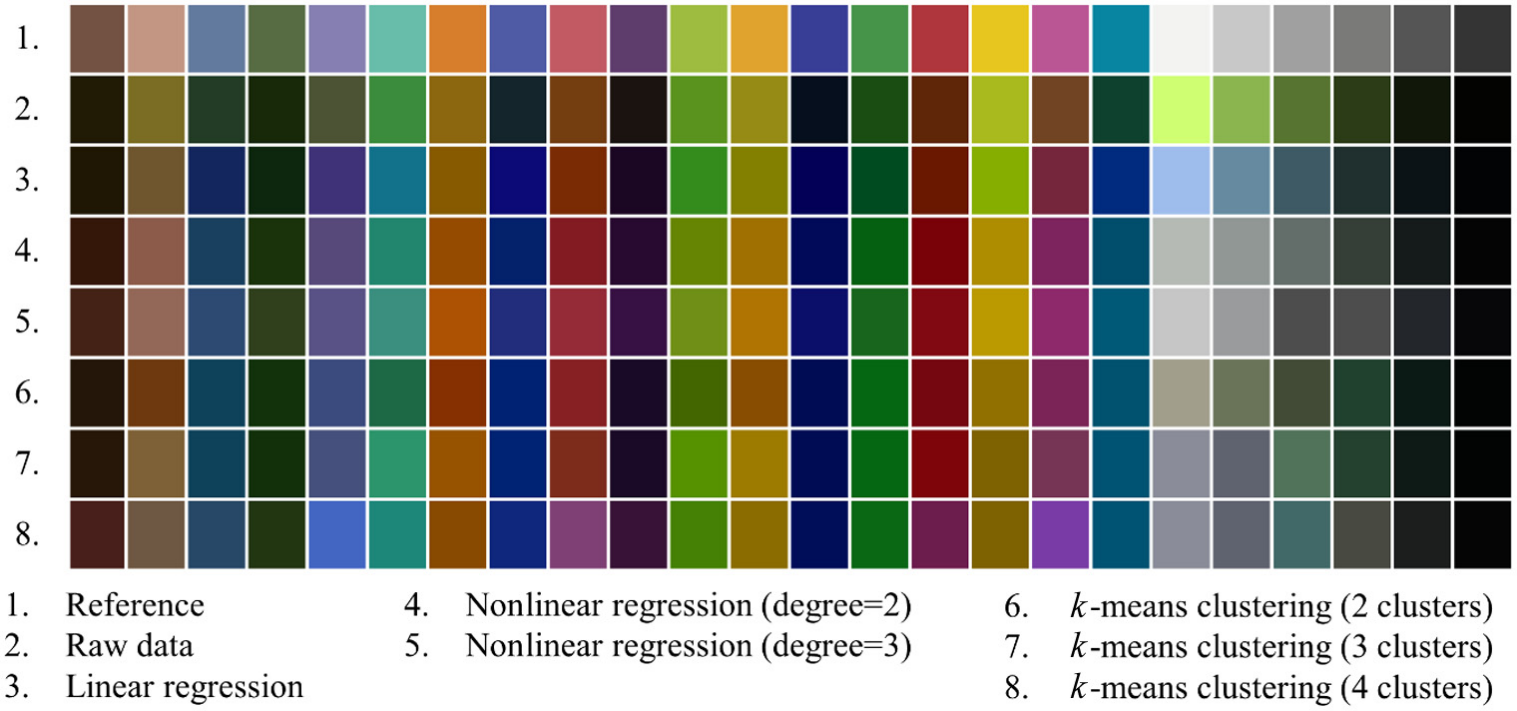
Comparison of color calibration methods for the 24 reference colors. Visual color assessment is in general only possible through a color-calibrated monitor.

**Fig. 13:**
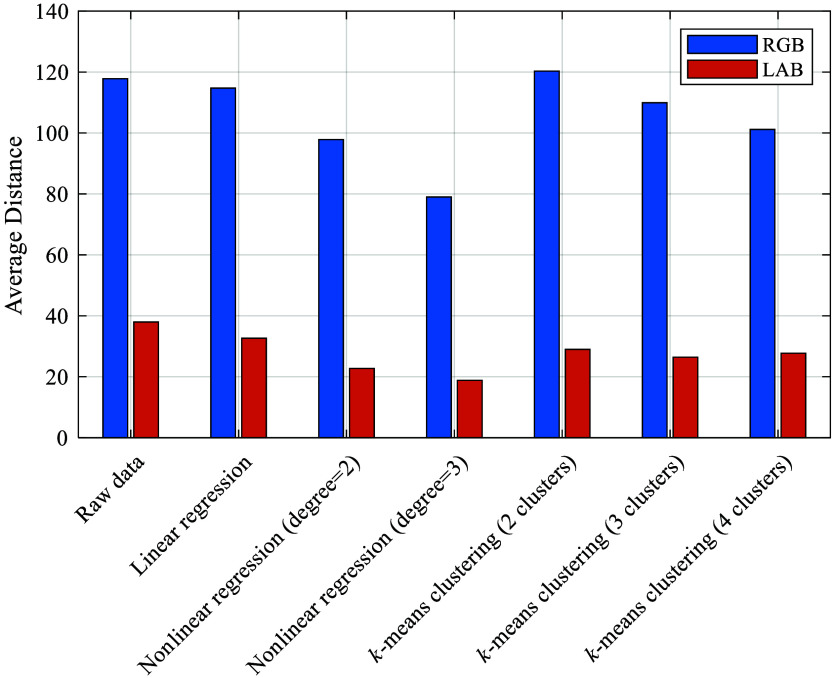
Objective comparison of the color calibrations based on color distance in the CIELAB and RGB color space. The color distances are averaged for all 24 colors.

Figure 12 illustrates the reference color values alongside the colors achieved through various calibration methods. The effectiveness of a color calibration can be assessed by measuring the absolute color distances from the reference values.^12^ However, prioritizing certain colors over others is challenging. Furthermore, the visual color assessment is only possible through a color-calibrated monitor. In addition, color comparisons are often still made subjectively using skin samples without a color-calibrated monitor.^12^ The chosen color calibration method significantly impacts the appearance of skin color, as demonstrated in Fig. 19 in the Appendix, which makes clear that the skin colors differ strongly across all methods. Figure 13 displays the average color distances in the RGB and CIELab spaces for each color calibration method.

**Fig. 14:**
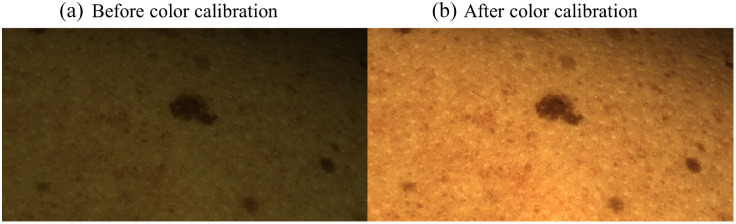
(a) Denoised image from the focus stack before color calibration. (b) Same image after color calibration, with the calibrated skin tone accurately matching the patient’s natural skin color. This step is part of the raw data preprocessing after image acquisition, see Fig. 4.

The comparison of the average distances to the reference colors reveals that the nonlinear regression with a degree of 3 has the smallest average distance to the reference colors in the CIELab and in the RGB color space. As a result of the analysis of the color calibration methods, the nonlinear regression method with a degree of 3 was used to calibrate the colors of the noncontact dermoscope in this work. Figure 14 shows a denoised raw image from the focus stack before (a) and after (b) the color calibration. This is part of the raw data preprocessing step after acquisition of the image stack, as indicated in Fig. 4. The color calibration of the imaging system is critical when the images are passed on to the dermatologist for the assessment. The top-right corner of the image after color calibration exhibits a skin region with less brightness. In future work, exposure bracketing could be implemented to achieve a more homogenous brightness distribution for such cases.^47^

### 3.4 Focus Stacking

**Fig. 15:**
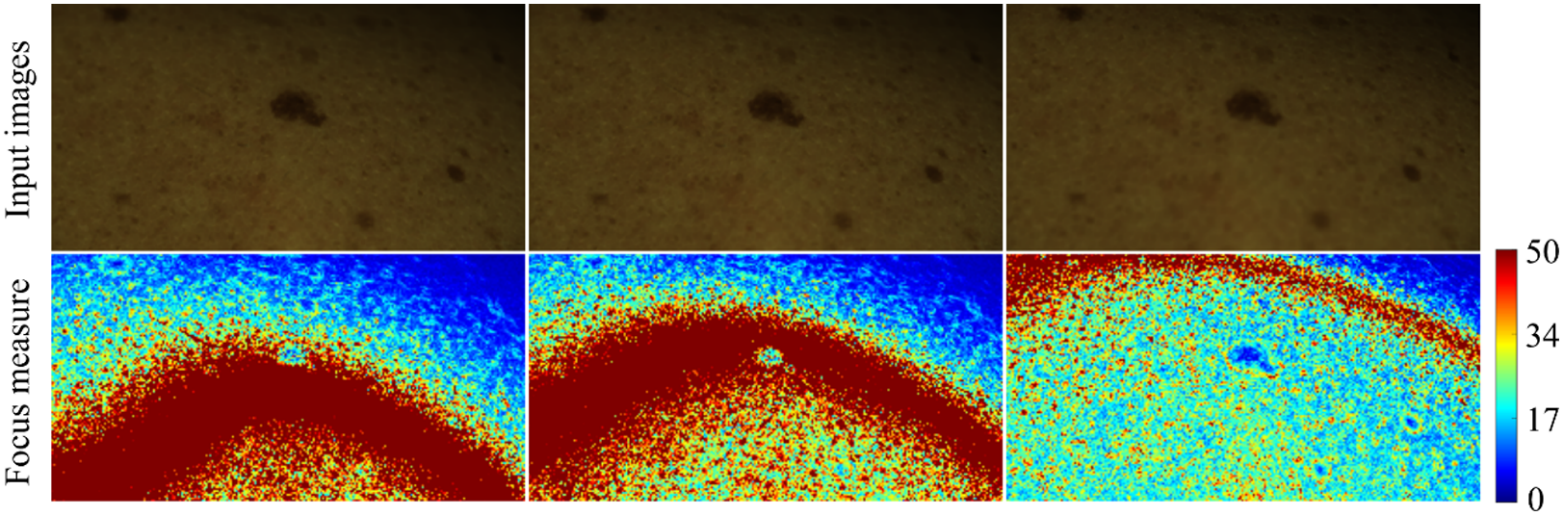
Top row: Three sample images from the image stack, each captured with different focus settings. Bottom row: Corresponding heatmaps where color intensity represents the degree of focus; warmer colors denote areas of higher focus in the image, whereas cooler colors indicate lower focus. The focus measure is presented as a dimensionless metric.

For focus stacking, image alignment is performed using the ECC method, as described in Sec. 2.5. The next step is to determine which areas of the acquired images of a lesion are in focus. This is achieved by computing a focus measure for each image. Figure 15 displays three exemplary images (upper row) from an image stack with varying levels of focus based on the Laplacian variance method. The accompanying heatmaps (lower row) illustrate the degree of focus in each image, with warmer colors indicating higher focus levels. By analyzing the focus measure heatmaps, it becomes evident that the dark red areas of each image are in sharp focus. This information is crucial for the subsequent image fusion process, where the goal is to combine the in-focus regions from each image into a single, fully focused composite image.

**Fig. 16:**
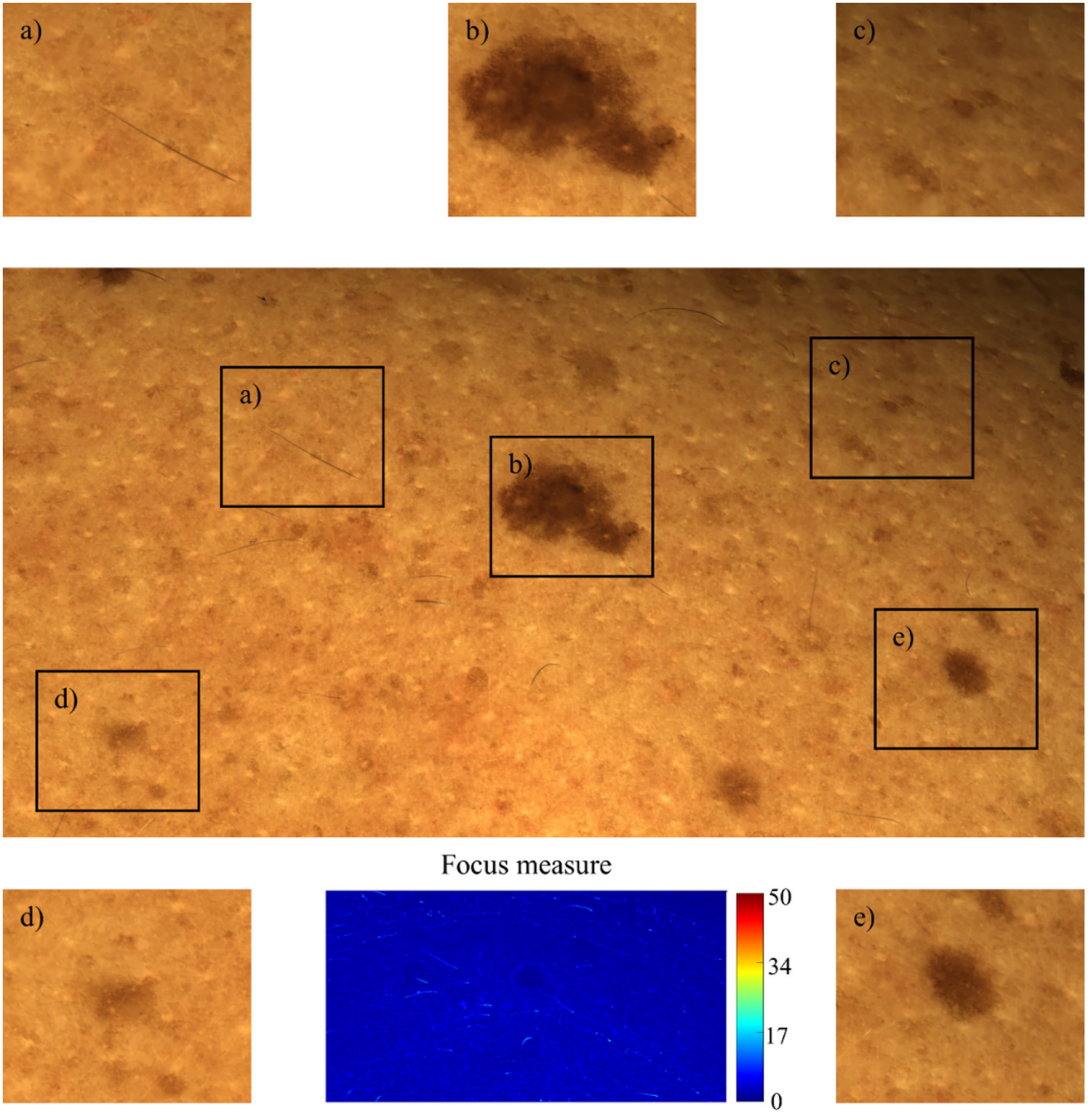
Result of the image fusion process. Cropped sections (a)–(e) highlight zoomed-in areas of the image, demonstrating uniform focus across the entire field. The center of the lower row displays the focus measure heatmap for the complete image, demonstrating a homogenous focus across the complete FOV.

The focus measure not only guides the selection of sharp regions but also serves as a weighting factor during the fusion, ensuring that the clearest parts of each image contribute most significantly to the result. This approach enhances the overall clarity and detail of the composite image, making it particularly useful in applications requiring precise and comprehensive visual information. Figure 16 shows the image fusion result for the image stack used as an example and the corresponding focus measure heatmap.

With the focus stacking method, we can combine multiple images from an image stack with varying degrees of focus over the images into a hyperfocus image with all skin areas in full focus. The image fusion assessment metrics described in Sec. 2.5 were used to validate that the focus stacking approach works reliably. This was done by computing the metric scores, which resulted in overall high values for our approach, also in comparison with other methods (for details of the procedure, see Ref. 13). The focus measure signal, now reduced to one-tenth of the local variance in the input images, indicates a more uniform distribution of focus. Although focus stacking can introduce a slight smoothing or averaging effect across the image, particularly in regions that were not consistently sharp across all inputs, this effect is minimal. The reduced focus measure values in the stacked image reflect the overall uniform sharpness, as the variance across the image decreases when all regions are in focus.

### 3.5 Super-Resolution

**Fig. 17:**
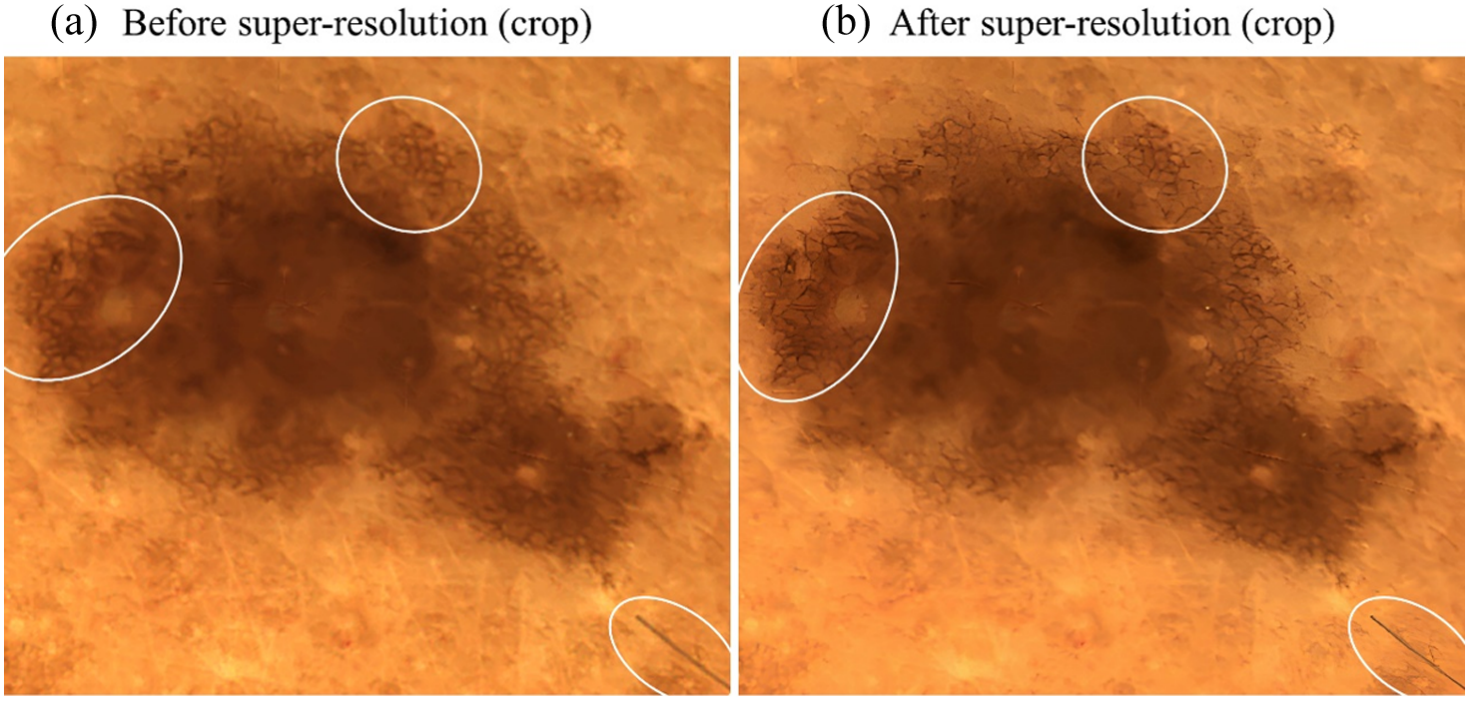
Cropped sections of the image shown in Fig. 16. (a) Section from the focus-stacked image. (b) Section after applying super-resolution with SRGAN. Highlighted regions demonstrate the impact of super-resolution on the pigment network, whereas the central lesion area appears visually unchanged by the process.

The complete focus-stacked image after applying the GAN-based super-resolution model described in Sec. 2.6 and cropped images of the main lesion in the image before and after application of the model are given in Fig. 17. From the comparison before and after super-resolution in Fig. 17, it is visible that the super-resolution technique is enhancing the resolution and contrast of the lesion and the hair. In particular, the pigment network exhibits an improvement in resolution and contrast, which is relevant for the application of classification algorithms in the next stage of our work. For the classification of malignant and benign lesions, particularly in the case of melanoma, it is crucial that the optical system resolves these structures, as also reported in Ref. 48.

### 3.6 Artificial Intelligence Assistance in Dermatology

**Fig. 18:**
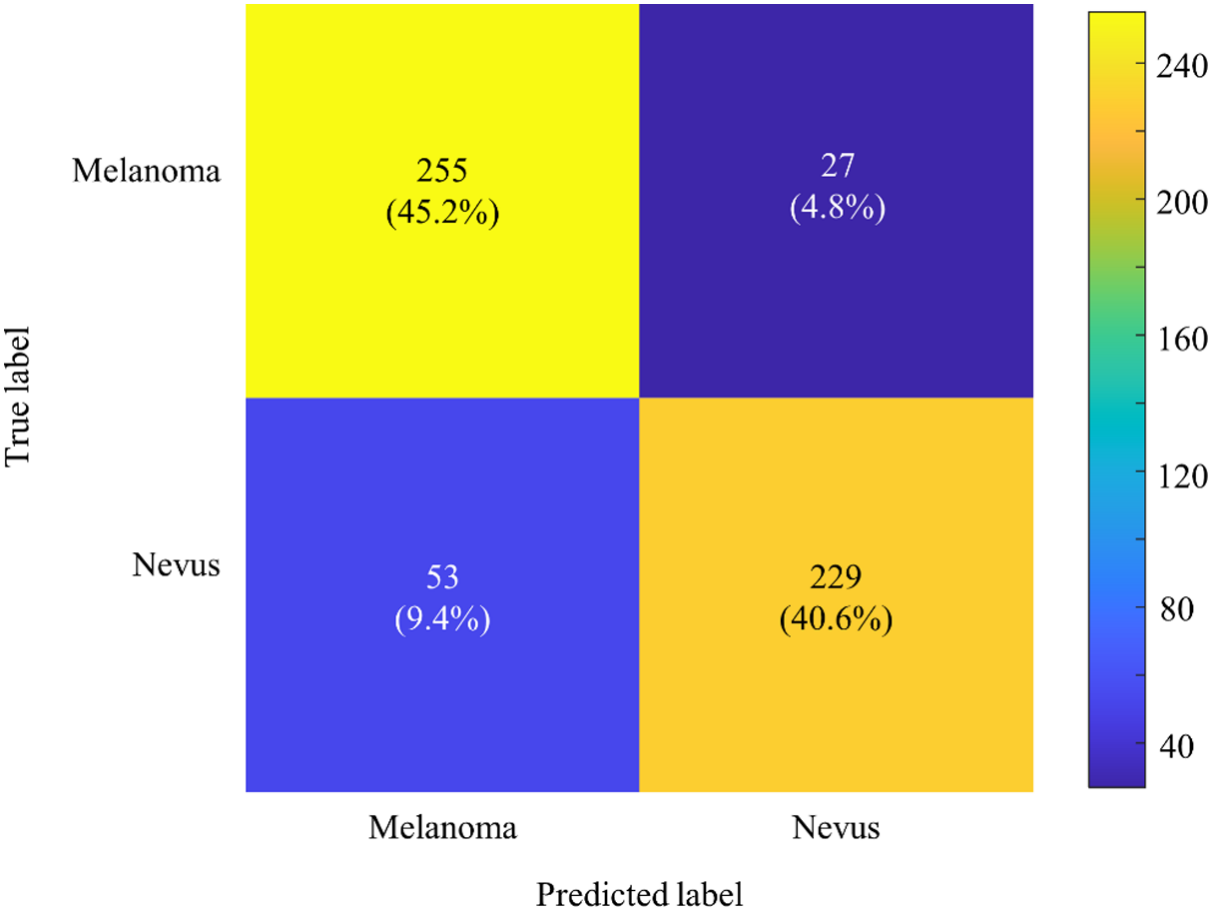
Confusion matrix for lesion classification with AutoKeras.

AI has shown significant potential in assisting dermatologists with lesion classification.^49^ This is particularly relevant for skin cancer screening, where early detection of melanoma is critical. In this study, we utilized an AutoKeras-based model to classify skin lesions into melanoma and nevus categories, with the performance of the model illustrated in the confusion matrix presented in Fig. 18. Images were selected as described in Sec. 2.8.

The confusion matrix demonstrates the distribution of true labels against predicted labels. In the context of melanoma detection, the primary objective is to minimize the number of false negatives, where the model incorrectly predicts a nevus when the actual condition is melanoma. In this scenario, false negatives (4.8%) are especially dangerous as they delay diagnosis and treatment, significantly impacting patient outcomes due to melanoma’s rapid progression and potential metastasis. Conversely, false positives (where a nevus is incorrectly classified as melanoma) account for 9.4% of cases in this model. Although false positives do not carry the same immediate risk as false negatives, they can still have negative consequences. Patients may experience unnecessary stress and undergo further invasive diagnostic procedures, such as biopsies or surgical excision, which could have been avoided. The ethical balance between false positives and negatives is thus crucial in AI-assisted diagnosis. The model applied in this work correctly identified 45.2% of all cases as melanoma and 40.6% of all cases as nevus, reflecting its overall capability in distinguishing between these two lesion types. However, these results highlight the importance of improving the model’s sensitivity and specificity to reduce the false negative rate. Achieving this is critical in making AI tools reliable and safe enough for real-world clinical applications, where missing a melanoma diagnosis has potentially life-threatening implications. Further refinements to the model can be achieved by employing more sophisticated training techniques, such as transfer learning, or by incorporating more comprehensive datasets to ensure the AI model is exposed to a broader range of lesion types. In addition, integrating AI-based predictions with expert dermatological review can serve as a hybrid approach to mitigate risks associated with misdiagnosis, combining the strengths of human expertise and AI’s efficiency in data analysis. Overall, the advancement of AI in dermatology holds great promise, but its implementation must be approached with caution, ensuring the highest standards of diagnostic reliability and patient safety.

## 4 Conclusion

In this work, we developed an AI-enhanced imaging system aimed at improving the early detection of melanoma through noncontact dermoscopy and hyperfocus imaging. The developed noncontact dermoscope can obtain dermoscopic images with full focus for all skin topographies by the implementation of focus stacking. The focus stacking procedure itself is implemented in the HRIM, based on an electrically adjustable focus realized by the electrically tunable liquid lens. By changing the current of the electromagnet in the liquid lens, differently focused images are captured in one sequence. The system’s electrically tunable liquid lens enables rapid image capture with a focus adjustment speed of 5  μs, keeping the acquisition time for a complete stack below 5 s. The HRIM operates at a capture rate of up to 50 FPS, ensuring efficient acquisition of high-resolution dermoscopic images and a resolution capability down to 28  μm. The most important pre-processing step is the color calibration. Because of the misalignment within the images of the stack, the images are aligned first with respect to each other, so that they can be overlaid. Extracting the in-focus area from each image is achieved by a local focus measure based on local variance. The focus measure is calculated for every pixel in the stack to evaluate its focus. Based on the calculated focus results, the images are fused to create an all-in-focus image.

The outcomes of this work are the developed HRIM, the algorithms, and implementation details to obtain skin images with the complete FOV in focus. We provide an easy-to-implement focus stacking–based approach to ensure all-in-focus images from a noncontact dermoscope. The developed classification model can assist the dermatologist in diagnosing a lesion.

Overall, the focus stacking delivers hyperfocus images of high resolution. The resolution of individual lesions is still below that of images acquired with standard contact dermoscopy. Super-resolution techniques based on deep learning are implemented to enhance the resolution and expose more structural details. Through our system, we achieved an all-in-focus FOV while maintaining the skin’s natural state, eliminating the need for contact dermoscopy. This advancement avoids the issues of skin distortion and infection risk inherent in contact methods.^9^ In addition, the integration of deep learning–based super-resolution algorithms has further enhanced the resolution and contrast of skin lesions, bringing them closer to the quality of contact dermoscopy. In future work, as soon as a large database of image stacks is acquired, the classification will be performed on the basis of the super-resolution images, similar to the method reported in Ref. 48, with a particular focus on reliable identification of melanoma. In addition, a quantitative criterion on feature enhancement for the features relevant for classification will be generated.

Another notable outcome of this work was the integration of an AutoKeras-based AI model designed to classify skin lesions as melanoma or nevus. Although the system demonstrates significant potential, the results highlighted critical areas for improvement in the model’s diagnostic performance, particularly in addressing false negatives and false positives. False negatives, where melanoma is misclassified as a benign nevus, occurred in 4.8% of cases. This is a crucial limitation, as missing a melanoma diagnosis can delay treatment and significantly impact patient outcomes due to the aggressive nature of melanoma. Reducing the false negative rate must be a priority to ensure the model’s clinical reliability and safety. Conversely, the system produced false positives in 9.4% of cases, where a nevus was incorrectly classified as melanoma. Although these errors are less life-threatening, they can lead to unnecessary patient anxiety, additional diagnostic procedures, and even unwarranted surgeries. Balancing the sensitivity to detect melanoma with specificity to avoid over-diagnosis is essential for the practical deployment of AI in clinical settings. The focus for future work will be on refining the AI model to lower the false negative rate through advanced training techniques, such as transfer learning with larger, more diverse datasets. By improving the model’s sensitivity and specificity, we aim to make it a safer and more reliable tool for dermatological diagnosis, complementing human expertise while reducing the likelihood of critical diagnostic errors. We will also focus on scaling the system to include multiple HRIMs for a full-body scanning solution and exploring new applications, such as integrating exposure bracketing, to achieve more uniform brightness.^50^ Future work will also attempt to introduce explainability of the AI classification^51^^–^^53^ and integrate and test an arrangement of multiple HRIMs in a fully automated total body scanning system with essentially real-time lesion assessment.^54^ Furthermore, we will focus on reducing false negatives in the classification algorithm, which requires a large database for different types of skin cancer to be examined. Another important task to be addressed in this respect is to ensure that all parts of the body, including curved skin surfaces, can be accessed with the imaging systems, which is intended to be achieved by mounting the systems on suitable robotic arms with sufficient degrees of freedom.

## 5 Appendix: Supplementary Material

### 5.1 Color Calibration

**Fig. 19:**
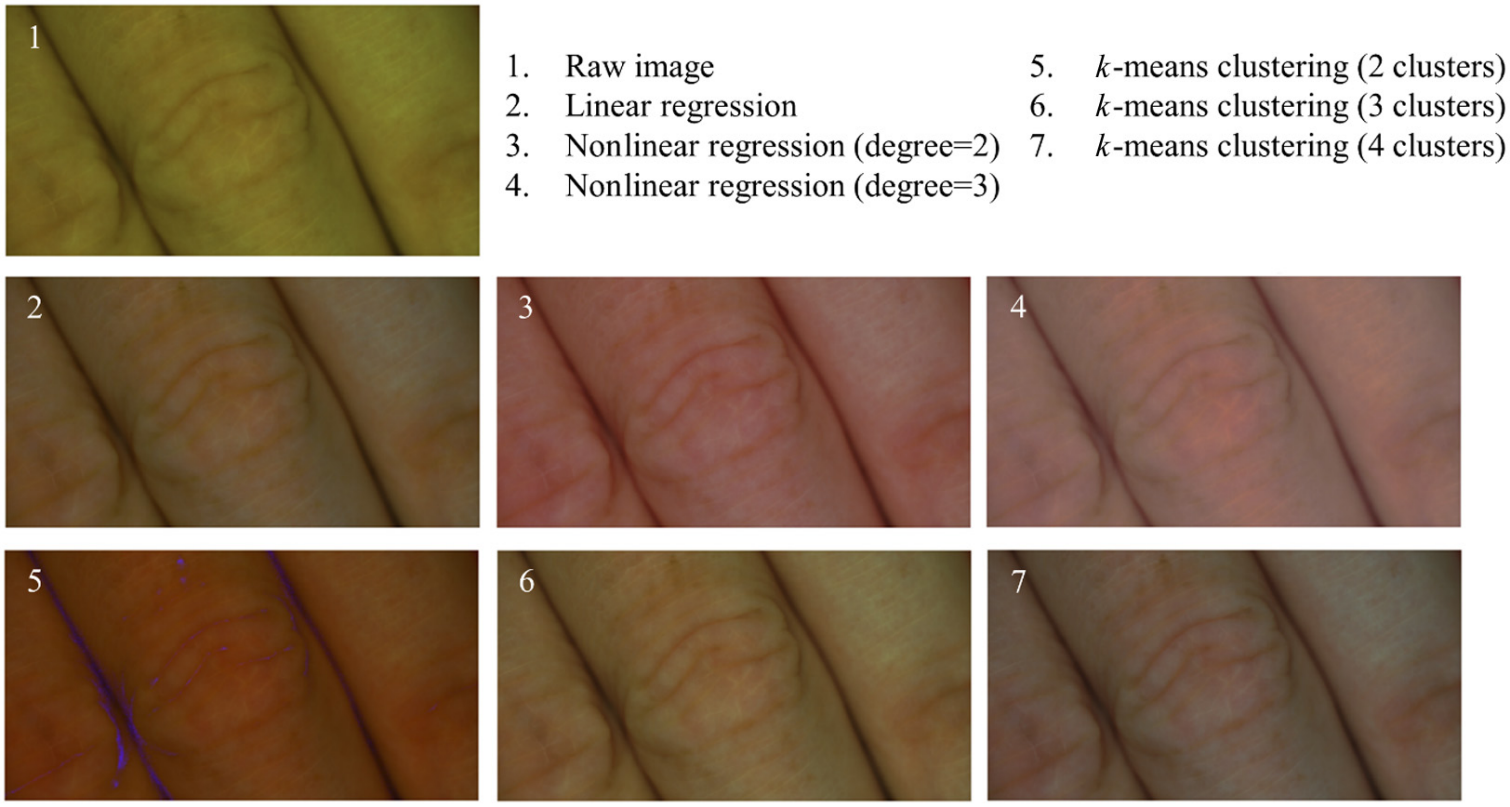
Comparison of the impact of color calibration methods on skin color appearance.

The finger sample in Fig. 19 was used to showcase the color calibration artifacts that can occur in the dark areas between the fingers, as is the case for the k-means clustering with two clusters. However, usually the images of the skin lesions are much more homogenous and do not exhibit such dark skin areas. The approach utilizing nonlinear regression with a degree of 3 yields the most natural-looking colors. Generally, an objective evaluation is needed to find the best color calibration.

## Data Availability

Code, data, and other materials are available upon reasonable request.
